# Comparing Oblique Lumbar Interbody Fusion with Lateral Screw Fixation and Transforaminal Full-Endoscopic Lumbar Discectomy (OLIF-TELD) and Posterior Lumbar Interbody Fusion (PLIF) for the Treatment of Adjacent Segment Disease

**DOI:** 10.1155/2020/4610128

**Published:** 2020-05-29

**Authors:** Zhuo Yang, Jianjun Chang, Lin Sun, Chien-Min Chen, Haoyu Feng

**Affiliations:** ^1^Shanxi Medical University, Taiyuan, Shanxi, China; ^2^Department of Orthopedics, Shanxi Bethune Hospital, Taiyuan, Shanxi, China; ^3^Division of Neurosurgery, Department of Surgery, Changhua Christian Hospital, Changhua, Taiwan; ^4^College of Nursing and Health Sciences, Da-Yeh University, Changhua, Taiwan; ^5^School of Medicine, Kaohsiung Medical University, Kaohsiung, Taiwan

## Abstract

**Background:**

A potential long-term complication of lumbar fusion is the development of adjacent segment disease (ASD), which may require surgical intervention and adversely affect outcomes. A high incidence of recurrent ASD was reported in patients who underwent the second (repeat) PLIF for symptomatic ASD. Herein, a feasible method, oblique lumbar interbody fusion combined with transforaminal endoscopic lumbar discectomy (OLIF-TELD) for dealing with adjacent lumbar disc herniation with upward or downward migration after lumbar spinal fusion, was proposed.

**Methods:**

A total of 19 patients who underwent revision surgery at ASD were consecutively enrolled. Clinical efficacy analysis included operative time, intraoperative bleeding, visual analogue scale (VAS) score, Oswestry dysfunction index (ODI) score, and Japanese orthopaedic association (JOA) assessment treatment score.

**Results:**

Among them, 11 patients were treated in a new surgical strategy, which is OLIF-TELD, and 8 patients underwent PLIF. There was no statistically significant difference between the two groups in terms of age, gender, and preoperative scores of VAS, ODI, and JOA. The operative duration was shorter, and intraoperative bleeding was less in the OLIF-TELD group compared with the PLIF group. PLIF had the greatest blood loss, and the OLIF-TELD group had lower VAS scores than the PLIF group postoperatively. The symptoms of all patients improved postoperatively with statistical significance.

**Conclusion:**

OLIF with lateral screw fixation combined with TELD may be an alternative surgical method for the treatment of adjacent lumbar disc herniation with upward or downward migration after lumbar fusion surgery.

## 1. Introduction

The number of lumbar fusions has increased and adjacent segment degeneration commonly develops following lumbar spine fusion [1–3]. Fusion surgery is a standard operative treatment for various pathologies of the lumbar spine with good clinical results. However, solid fusion has been reported to accelerate degenerative changes at adjacent unfused levels. A potential long-term complication of lumbar fusion is the development of adjacent segment disease (ASD), which may require surgical intervention and adversely affect outcomes. ASD is defined as new degenerative changes from a spinal level adjacent to a surgically treated level or levels in the spine, accompanied by related symptoms (radiculopathy, myelopathy, or instability) [4]. ASD is considered any abnormal process that develops in the mobile segment next to a spinal fusion [5]. The incidence of ASD development after lumbar surgery and required additional surgery was from 5.0 to 15.0% [6, 7]. Potential risk factors for developing ASD after lumbar fusion include instrumentation, fusion length, sagittal malalignment, facet injury, age, and preexisting degenerative changes [8]. The current treatment of ASD is achieved by decompression and fixation or expansion of existing structures. This was done through a posterior approach decompression, interbody fusion, and pedicle screw fixation. Posterior lumbar interbody fusion (PLIF) is one of the standard treatments for unstable vertebral disorders and allows for neural decompression [9]. However, a degenerative change at unfused adjacent levels, nerve roots injury, and chronic radiculopathy has been reported as disadvantages of PLIF. A high incidence of recurrent ASD (44%) in patients who underwent the second (repeat) PLIF for symptomatic ASD was reported [10]. Apart from PLIF, the main methods of spinal fusion include transforaminal lumbar interbody fusion (TLIF) and anterior lumbar interbody fusion (ALIF). However, they all have their weaknesses and limitations. To overcome the obstruction of the approaches of ALIF and lateral lumbar interbody fusion (LLIF), the oblique lumbar interbody fusion (OLIF) was proposed to enter the lumbar spine through the interspace between the abdominal aorta and the psoas major muscle. OLIF has been reported being a safe procedure with a high fusion rate based on CT findings, and the complications are acceptable when compared with LLIF and ALIF [11]. Transforaminal endoscopic lumbar discectomy (TELD) is one of the most popular minimally invasion spine surgeries and mainly used for lumbar disc herniation treatment from a lateral approach [12]. TELD for treatment of symptomatic lumbar disc herniation can result in better outcomes, the small size of the incision, the lower rate of complications, and shorter hospitalization time [13]. A variety of surgical solutions have emerged for ASD. Herein, we propose a feasible method for the treatment of adjacent lumbar disc herniation after lumbar spinal fusion, which combines multiple minimally invasive operations, oblique lumbar interbody fusion (OLIF) with lateral screw fixation for indirect decompression, and transforaminal endoscopic lumbar discectomy (TELD) for direct decompression. Without the destruction of the lumbar spine posterior structure, the recurrence of ASD can be avoided. The effectiveness of oblique lumbar interbody fusion (OLIF) with lateral screw fixation and transforaminal endoscopic lumbar discectomy (TELD) for dealing with adjacent lumbar disc herniation after lumbar spinal fusion was evaluated. Besides, the efficacy in the treatment of adjacent lumbar disc herniation between OLIF-TELD and PLIF was compared.

## 2. Material and Method

**Figure 1:**
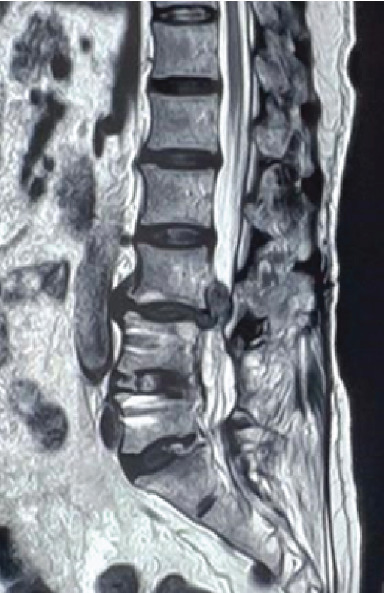
Lumbar MRI shows L3-4 adjacent segment degeneration and lumbar disc herniation with upward migration.

This is a retrospective study, and informed consent was obtained from all patients. Patients were prospectively entered into this study, and all clinical data were collected through medical records reviewed retrospectively. ASD was defined as both a radiographically and clinically significant disease at a level adjacent to a previous fusion requiring surgical intervention. Patients were included if they had met all the following indications including radicular pain, lumbar instability, lumbar disc herniation with upward or downward migration, and mild to moderate lumbar spinal canal stenosis. TELD allows for direct decompression and can be used for the treatment of upward or downward migrated lumbar disc herniation. To ASD, adjacent lumbar disc herniation with upward or downward migration was confirmed by magnetic resonance imaging (MRI) (Figure 1). All of them met the inclusion criteria and were treated with either OLIF combined with TELD or PLIF alone for ASD. All surgical procedures were performed by the same surgeon. The exclusion criteria included cauda equina syndrome and coexistent pathologic conditions, such as infection and tumor. From September 2017 to April 2019, a total of 19 patients who underwent revision surgery at ASD were consecutively enrolled. Among them, 11 patients underwent OLIF combined TELD and 8 patients underwent PLIF.

## 3. Operation Procedures

### 3.1. Transforaminal Full-Endoscopic Lumbar Discectomy (TELD)

**Figure 2:**
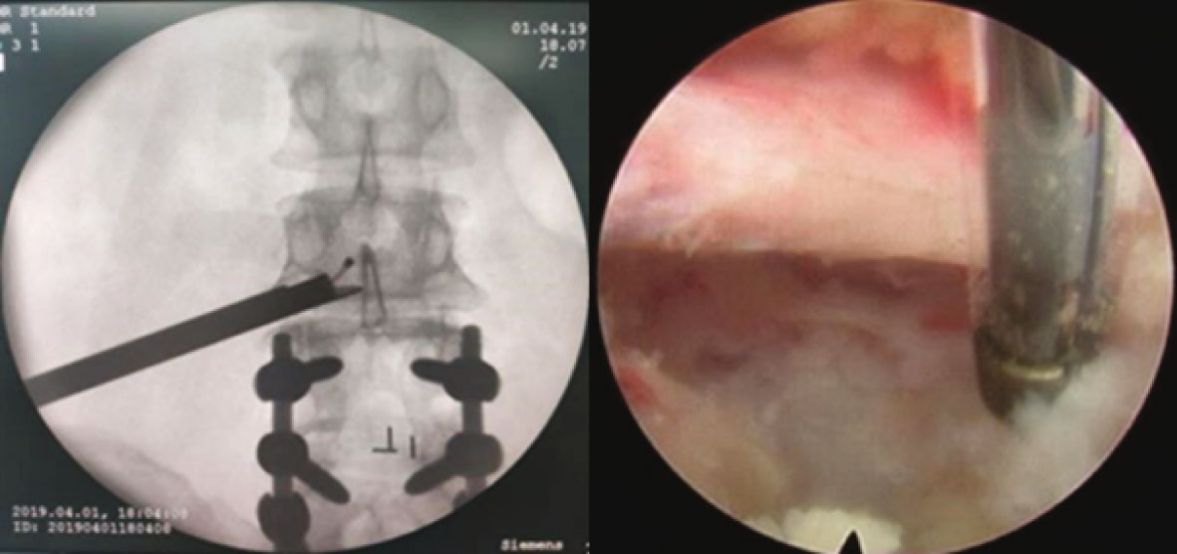
Intraoperative fluoroscopic images showing direct decompression under endoscopic view.

All patients were placed in the prone position on an operating table and under local anesthesia. The point of entry should be approximately 10-14 cm lateral from the posterior midline. The guiding needle was punctured into the vertebral superior articular process, which is a safe area at the angle which was about from 15 to 30-degree angle, and a guidewire was put on the needle. A skin cut (8 mm) was made, and the incision was dilated by a dilator and working channel. A 7.9 mm diameter working sleeve was inserted in sequence. Then, the dilator was removed and the spinal endoscope was inserted (SPINENDOS GmbH, Munich, Germany). The operation was performed via the intraendoscopic working channel using alternating sets of instruments under full visual control and continuous normal saline irrigation. Then, the light source was opened, and the herniated disc tissue and the free nucleus pulposus were removed by a foraminoplastic technique using a high-speed drill (SPINENDOS GmbH, Munich, Germany) and different types of nucleus pulposus forceps. At the same time, a bipolar radiofrequency (SPINENDOS GmbH, Munich, Germany) was used for shaping the fiber ring and hemostasis. The endoscopic working sleeve was removed after the decompression was completed. The patient underwent TELD is shown in Figure 2.

### 3.2. Oblique Lumbar Interbody Fusion (OLIF)

**Figure 3:**
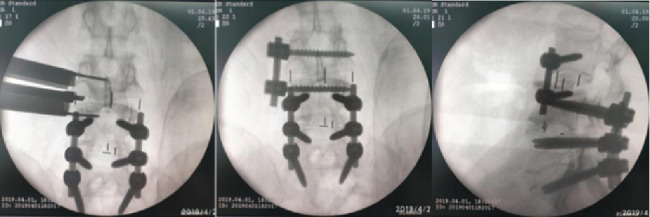
Radiographic images showed the surgical process of OLIF with lateral screw fixation.

After general anesthesia, the right lateral position was taken, the left leg was slightly flexed, and the operating bed was adjusted to make the lumbar-like bridge which can fully expose the target segment. The target segment was marked under C-arm guidance. About 7-8 cm skin incision was made according to the directions of the fibers of external oblique muscle, internal oblique muscle, and transverse abdominal muscle. Blunt dissection was performed in the space which was in the retroperitoneal space between the abdominal aorta and the psoas major muscle. A ball of gauze and the index finger were used to push the psoas major muscle and the sympathetic trunk to the back. The channel is placed and fixed on the channel nail and the free arm, after using a guide needle and dilator, the light source was connected. After discectomy and the osseous endplate was exposed, a suitable cage for interbody fusion was inserted under fluoroscopic control, which was filled with artificial bone and restorative materials. Lateral screws were placed on the lateral side of the vertebral body to make the screws pass through the contralateral cortex as far as possible, the operating table was recovered, and the rod was installed. Finally, the incision was closed layer by layer. The patient who underwent OLIF is shown in Figure 3.

### 3.3. Posterior Lumbar Interbody Fusion (PLIF)

**Figure 4:**
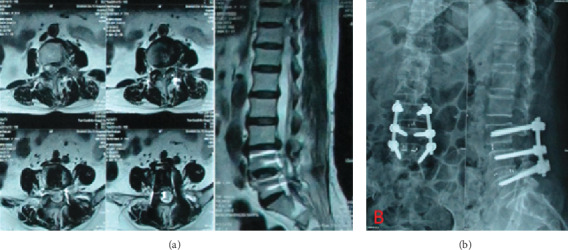
A case of PLIF surgery. (a) Preoperative Lumbar MRI images show L3/L4 intervertebral disk prolapse. (b) Postoperative frontal and lateral X-ray images.

To begin the procedure, the patient was placed in a prone position after general anesthesia. The original incision scar was incised; all previously implanted nuts and screw rods were exposed and removed, then the rostral adjacent vertebra was exposed, and 2 pedicle screws were inserted. Bilateral laminotomies and medial facetectomies were performed using a rongeur; subsequently, the thecal sac and nerve roots were exposed. A scalpel blade was used to make bilateral annular windows, and the nucleus pulposus was removed. A thorough discectomy was performed using a combination of shavers, curettes, and rongeurs to expose the bony endplate. The autologous bone was then implanted into the disc space, and an appropriate size cage packed with bone autograft was inserted. Bilateral pedicle screws were connected with new elongated screw rods and fixed with nuts. A drainage tube was placed in the wound, and the incision was closed. A typical case of PLIF is shown in Figure 4.

### 3.4. Outcome Evaluation

Clinical outcomes were evaluated using the visual analog scale (VAS; ranging from 0 to 10), score of pain, Oswestry Disability Index score (ODI; ranging from 0 to 100, and higher scores mean more severe disability), and the Japanese orthopaedic association assessment treatment score (Japanese orthopaedic association scores, JOA; ranging from 0 to 29 and lower scores mean the more severe the deficits) preoperatively and postoperatively at 1 week and 3 months. The clinical variables include preoperative comorbidities, operation time, and disc levels of decompressed and intraoperative bleeding.

### 3.5. Statistical Analysis

Data are expressed as the mean ± standard deviation for continuous variables. Mann–Whitney *U* test was used for continuous variables. A paired *t*-test was performed to compare the mean differences between two-time intervals on pain relief before and after lumbar surgery. Fisher's exact test was used in the analysis of categorical variables. A *p* value < 0.05 was considered to indicate statistical significance, and all tests were two-tailed. All statistical analyses were performed on a personal computer with the statistical package SPSS for Windows SPSS (Version 16.0, Chicago, IL, USA).

## 4. Results

Patients were aged 62.79 ± 12.16 years. The OLIF-TELD approach was done in 11 patients. The procedure was performed at L3–5 in 2, L4–5 in 6, and L4-S1 in 3 patients. The PLIF approach was done in 8 patients. The procedure was performed at L1-5 in 1, L2-S1 in 1, L3–5 in 1, L4-S1 in 4, and L5-S1 in 1 patient (Table 1). There was no statistically significant difference between the two groups in terms of age, gender, and preoperative scores of VAS, ODI, and JOA. No significant differences in ODI and JOA were found at follow-up time points between the two groups. The operative duration was shorter (127.27 ± 21.49 v.s. 204.38 ± 101.82 minutes; *p* = 0.032) and intraoperative bleeding (115.45 ± 19.16 v.s. 737.50 ± 501.25 *ml*; *p* = 0.003) was less in the OLIF-TELD group compared with the PLIF group. PLIF had the greatest blood loss, and the OLIF-TELD group had lower VAS scores than the PLIF group postoperatively (*p* = 0.001) (Table 2). The symptoms of all patients improved postoperatively with statistical significance (Table 3). In the OLIF-TELD group, the preoperative mean JOA score was 10.36 ± 6.19 and increased to 19.82 ± 4.79 postoperatively (*p* = 0.003). The mean VAS score was 5.27 ± 2.83 preoperatively and 1.73 ± 1.35 postoperatively (*p* = 0.007). The mean ODI score improved from 60.68 ± 23.99 to 31.92 ± 14.78 (*p* = 0.007). In the PLIF group, the preoperative mean JOA score was 10.75 ± 1.67 and increased to 19.00 ± 2.00 postoperatively (*p* = 0.011). The mean VAS score was 7.12 ± 1.25 preoperatively and 4.12 ± 0.99 postoperatively (*p* = 0.011). The mean ODI score improved from 73.89 ± 8.38 to 41.95 ± 13.98 (*p* = 0.012).

## 5. Discussions

In recent years, the number of spinal fusions for lumbar spine diseases significantly increased and is associated with an increased incidence of ASD. Traditional open PLIF has been reported to cause unavoidable damage to paraspinal muscles, soft tissue, and posterior bone structure of the lumbar spine [14]. The occurrence of ASD is the result of a variety of factors. The risk factors for ASD after lumbar fusion surgery included age, genetic factors, high body mass index, preexisting adjacent segment degeneration, laminectomy at the adjacent level of fusion, the excessive distraction of the fusion level, insufficient lumbar lordosis, multilevel fixation, floating fusion, coronal wedging of L5-S disc, pelvic tilt, and osteoporosis. Preserving the posterior element of the fusion site has less risk of ASD than conventional fusion surgery [15]. Although a variety of factors related to the incidence of ASD has been reported, but which factors account for a large proportion are still controversial. It has been reported that patients who had initial preoperative adjacent disc degenerative changes and short fixation are important risk factors in the development of ASD after lumbar fusion [16]. OLIF has relatively broad indications with a low risk of vascular complications and neurologic complications [17]. Through the review of the literature, the risk factors for ASD after lumbar fusion have been addressed. Patients with increased BMI, preoperative adjacent disc degeneration (ADD), disc collapse, disc bulge in MRI, and a smaller preoperative relative cross-sectional area (CSA) of the paraspinal muscle on MRI have a statistically significant increased risk of developing symptomatic ASD after posterior lumbar fusion [18–20]. Posterior open surgery was the main surgical method for ASD previously. This type of surgery as well as PLIF and TLIF allows for directly decompression and stabilization of the fused segment. However, they had great damage to the facet joints, spinous processes, and ligaments of the lumbar vertebrae. During reoperation, the extensive scar after the first operation will lead to a significant increase in the difficulty and risk of reoperation, and there is a risk of recurrence of ASD after reoperation. According to the literature about the revision of spinal fusion for the management of ASD, the effective use of minimally invasive surgical transforaminal lumbar interbody fusion (MIS-TLIF) [21] and lateral lumbar interbody fusion (MIS-LLIF) [22] in ASD treatment has been reported. Besides, it has been concluded that MIS-TLIF/PLIF can reduce the incidence rate of ASD, compared with open surgery [23]. Minimally invasive OLIF can be performed safely in the lumbar spine from L1 to L5 and at L5-S1. The complication profile appears acceptable when compared with LLIF and ALIF [11, 24]. Lateral screw fixation through the OLIF incision can avoid changing the position of operation, incision again, the injury of the spinal cord, and nerve by pedicle screw. The operation achieved the goal of indirect decompression by broadening the intervertebral height and intervertebral foramen area. Compared with MIS-TILF, this technique does not need to remove the paravertebral muscles during the operation. Compared with LLIF, less injury of the lumbar plexus in the psoas major muscle is caused, and there was no need for nerve monitoring during operation. ALIF was more suitable for the fusion of the L5-S1 segment.

Regarding ASD treatment after lumbar fusion, it has been reported that the clinical outcomes of using a TELD procedure to treat single-level ASD were similar to those of the PLIF approach. However, the TELD procedure had significant advantages in small incision, low risk of posttreatment trauma, shorter hospitalization time, and less blood loss [25]. For decompression, OLIF can provide indirect decompression, but the degree of its decompression is limited, which limits its application to a certain extent. The approaches of OLIF and TELD are different and do not affect each other. We combine OLIF and TELD [26] for decompression and fusion of ASD. TELD may augment the indirect decompression effect of OLIF, and OLIF can provide a satisfactory fusion rate, so they can make up for each other's deficiencies. Although the stability of lateral fixation was not as stable as that of the percutaneous pedicle screw, but it could reduce the torsion and lateral bending motion and had some effect on the fixation of adjacent segmental lesions. However, the main purpose of this fixation method is to promote fusion of the target segment and prevent the displacement of the cage to achieve osseous fusion between the vertebrae.

## 6. Conclusion

OLIF with lateral screw fixation combined with TELD may be an alternative surgical method for the treatment of adjacent lumbar disc herniation with upward or downward migration after lumbar fusion surgery. The operation has less trauma, less complications, and a definite short-term curative effect. However, due to the small sample size, inadequate imaging data measurement, and short follow-up time, a multicenter randomized controlled study with a larger sample size is needed to further evaluate its long-term efficacy.

### 6.1. Limitation

The present study also has some limitations. First, the data were collected at a single medical center in China, which may somewhat limit the applicability of the study results. Second, the inclusion criteria were rather restrictive and may have introduced selection bias. Third, the follow-up period was short. Prospectively, cohort studies with more patients and longer follow-up time are needed to further verify the efficacy of OLIF with TELD in treating ASD.

## Figures and Tables

**Table 1:** Demographics data of patients in OLIF-TELD and PLIF group.

		OLIF-TELD (*n* = 11)	PLIF (*n* = 8)	*p*	Total (*N* = 19)
Age		62.73 ± 11.78	62.88 ± 13.49	0.649	62.79 ± 12.16
Gender (%)					
	Female	4 (36.4)	5 (62.5)	0.370	9 (47.4)
	Male	7 (63.6)	3 (37.5)		10 (52.6)
The level of fusion					
L1-5		0	1	0.100	1
L2–S1		0	1		1
L3–5		2	1		3
L4-5		6	0		6
L4–S1		3	4		7
L5-S1		0	1		1

Note. OLIF-TELD: oblique lumbar interbody fusion-transforaminal endoscopic lumbar discectomy; PLIF: posterior lumbar interbody fusion.

**Table 2:** Comparisons of VAS, ODI, and JOA scores between OLIF-TELD and PLIF groups.

	OLIF-TELD (*n* = 11)	PLIF (*n* = 8)	*p*
Operative duration, min	127.27 ± 21.49	204.38 ± 101.82	0.032
Intraoperative hemorrhage, mL	115.45 ± 19.16	737.50 ± 501.25	0.003
Preoperative VAS	5.27 ± 2.83	7.12 ± 1.25	0.070
Preoperative ODI, %	60.68 ± 23.99	73.89 ± 8.38	0.302
Preoperative JOA	10.36 ± 6.19	10.75 ± 1.67	0.229
Postoperative VAS	1.73 ± 1.35	4.12 ± 0.99	0.001
Postoperative ODI, %	31.92 ± 14.78	41.95 ± 13.98	0.262
Postoperative JOA	19.82 ± 4.79	19.00 ± 2.00	0.803

Note. OLIF-TELD: oblique lumbar interbody fusion-transforaminal endoscopic lumbar discectomy; PLIF: posterior lumbar interbody fusion; VAS: visual analog scale; ODI: Oswestry disability index; JOA: Japanese orthopaedic association.

**Table 3:** Comparisons of VAS, ODI, and JOA scores in patient with either OLIF-TELD or PLIF before and after operation.

	OLIF-TELD (*n* = 11)	*p*	PLIF (*n* = 8)	*p*
Preoperative VAS	5.27 ± 2.83	0.007	7.12 ± 1.25	0.011
Postoperative VAS	1.73 ± 1.35		4.12 ± 0.99	
Preoperative ODI, %	60.68 ± 23.99	0.003	73.89 ± 8.38	0.012
Postoperative ODI, %	31.92 ± 14.78		41.95 ± 13.98	
Preoperative JOA	10.36 ± 6.19	0.003	10.75 ± 1.67	0.011
Postoperative JOA	19.82 ± 4.79		19.00 ± 2.00	

Note. OLIF-TELD: oblique lumbar interbody fusion-transforaminal endoscopic lumbar discectomy; PLIF: posterior lumbar interbody fusion; VAS: visual analog scale; ODI: Oswestry disability index; JOA: Japanese orthopaedic association.

## Data Availability

The data that support the findings of this study are available from the corresponding author, HF, upon reasonable request.
